# The effect of root orientation on inferior alveolar nerve injury after extraction of impacted mandibular third molars based on propensity score-matched analysis: a retrospective cohort study

**DOI:** 10.1186/s12903-023-03661-0

**Published:** 2023-11-27

**Authors:** Shijun Kuang, Yuhao Liu, Weijie Zhuang, Kechen Li, Wenbin Yang, Yihong Tian

**Affiliations:** 1 Hospital of Stomatology, Guangdong Provincial Clinical Research Center of Oral Diseases, Guangdong Provincial Key Laboratory of Stomatology, Guangdong Key Laboratory for Dental Disease Prevention and Control, Sun Yat-Sen University, No.56, Lingyuan West Road, Yuexiu District, Guangzhou City, Guangdong Province 510030 China; 2 State Key Laboratory of Oral Diseases, National Clinical Research Center for Oral Diseases, West China Hospital of Stomatology, Sichuan University, No.14, Third Section of Ren Min Nan Road, Chengdu City, Sichuan Province 610041 China

**Keywords:** Propensity score matching (PSM), Impacted mandibular third molars (IMTMs), Inferior alveolar canal, Inferior alveolar nerve, Cone beam computed tomography (CBCT)

## Abstract

**Background:**

The injury of the inferior alveolar nerve (IAN) is one of the most serious complications of impacted mandibular third molars (IMTMs) extraction. The influence of the root orientation of IMTMs on IAN injury is still controversial. A deeper understanding of the risk factors of IAN injury conduces to better prevention of IAN injury. This study aims to explore whether root orientation is an independent risk factor of IAN injury during IMTMs extraction using the statistical strategy of propensity score matching (PSM).

**Methods:**

This retrospective cohort study included 379 patients with 539 cases of high-risk IMTMs screened by panoramic radiography and cone beam computed tomography. The IAN injury incidence after extraction of different groups of IMTMs was analyzed using the chi-square test or Fisher’s exact test. The correlation between third molar root orientation and impaction depth/contact degree with IAN was evaluated by the Lambda coefficient. Based on PSM for balancing confounding factors including age, sex, impaction depth, and contact degree, the effect of root orientation on the incidence of IAN injury was further analyzed using Fisher’s exact test.

**Results:**

There were significant group differences in IAN injury incidence in impaction depth, root orientation, and contact degree of root-IAC before PSM. Root orientation was correlated with impaction depth and contact degree of root-IAC. After PSM, there were 9 cases with IAN injury and 257 cases without IAN injury. There were significant group differences between the buccal and non-buccal groups after PSM, and the risk of IAN injury was higher when the root was located on the buccal side of IAC (OR = 8.448, RR = 8).

**Conclusions:**

Root orientation is an independent risk factor of IAN injury, and the risk is higher when the root is located on the buccal side of IAC. These findings could help better evaluate the risk of inferior alveolar nerve injury before the extraction of IMTMs.

**Supplementary Information:**

The online version contains supplementary material available at 10.1186/s12903-023-03661-0.

## Background

The injury of the inferior alveolar nerve (IAN) is one of the most serious complications after extraction of impacted mandibular third molars (IMTMs), with an incidence of 0.4% to 20% [1]. IAN injury generally causes numbness or pain in the chin skin, lower lip, and lower corner of the mouth that are innervated by IAN, which has a significant effect on the patient's physical and mental health [2, 3]. In addition to subjective factors such as the doctor's experience [4], the patient's demographic conditions (such as age and sex) and the impaction features of IMTMs both significantly affect the incidence of IAN injury. A deeper understanding of the risk factors of IAN injury conduces to better prevention of IAN injury [5].

Based on radiological analysis of IMTMs, previous studies have revealed that the impaction depth of IMTMs and the contact degree of the IMTM root with the inferior alveolar canal (IAC) significantly affect the incidence of IAN injury after extraction of IMTMs. However, the influence of the root orientation of IMTMs is controversial [6–9]. This may be attributed to the fact that most of the previous relevant studies were observational and thus were vulnerable to biases and confounding factors [10]. The contact degree of root-IAC, impaction depth, age, and sex, may vary significantly among IMTMs with different root orientations. Since univariate analysis cannot eliminate the interference of these confounding factors, the comparability between different groups cannot be guaranteed, which may even lead to contradictory conclusions. To determine whether the root orientation of IMTMs is an independent risk factor of IAN injury after extraction of IMTMs, reduction in characteristics differences of the subjects themselves necessitates a more reasonable statistical strategy.

Propensity score analysis is a statistical methodology introduced by Rosenbaum and Rubin DB which is adopted in many clinical studies in recent years [11, 12], owing to the advantage that it can balance the distribution of confounding factors and thus provide a better insight into the effect of a single variable on the outcome [13]. Its principle is to model through binary/multivariate logistic regression, to calculate the estimated probability of each patient assigned to a specific exposure group, and to convert the given confounding factors into a propensity score. By means such as nearest neighbor matching and optimal matching, subjects with similar scores in a specific group and the control group are matched at the ratio of 1:1 or 1:n, that is, propensity score matching (PSM) [14].

To our knowledge, there is a lack of application of PSM to the analysis of risk factors of IAN injury after extraction of IMTMs. Based on respective analysis of the effect of impaction depth, root orientation, and contact degree of root-IAC, and confounding factor balancing by PSM, this study is aimed to further analyze the effect of root orientation of IMTMs on the incidence of IAN injury after extraction of IMTMs. By verifying whether root orientation is an independent risk factor, this study provides new evidence for reasonable prediction of IAN injury after extraction of IMTMs.

## Methods

### Study design and patient recruitment

The sample size is set according to the previous literature [15–17]. In this study, the sample size is originally set to 300 people. After the data analysis, we used the software PASS v15 (Power Analysis and Sample Size) to verify the sample size of this study. Using the “Tests for Two Proportions” in the software, we set α to 0.05 and β to 0.2, then input the incidence of IAN injury of the buccal group (P1), the incidence of IAN injury of the non-buccal group (P2) and the ratio of the case number of the two groups (N2/N1) into the software. The result shows that the total sample size needed for the study is 449.

This study is a retrospective cohort study with a follow-up period of 6 months. Patients with IMTMs extracted in the department of oral and maxillofacial surgery of the Hospital of Stomatology, Sun Yat-sen University, from December 2021 to November 2022, were screened in this study. The IMTMs with root-IAC contact were preliminarily screened by panoramic radiography and were further diagnosed by cone beam computed tomography (CBCT). A total of 539 IMTMs, including 268 IMTMs from the left side and 271 IMTMs from the right side, were chosen from 379 patients that included 124 males and 255 females and featured an average age of 29.1 years old (18 ~ 59 years old). The complete table of all IMTMs is shown in Supplementary Table S1. Considering that IAN dominates unilateral sensory and IAN injury is dependent on unilateral tooth extraction, each tooth was independently numbered for statistical analysis. Epidata v3.1 was used for sample data entry, and Microsoft Excel 2021 was used for data integration.

Inclusion criteria: (1) the patient having complete data of panoramic radiography and CBCT, (2) the imaging of CBCT showing root-IAC contact, (3) The patient met the indications for impacted mandibular third molar extraction, having no contraindication for tooth extraction and successful tooth extraction. (Refer to Supplementary Table S2 for indications and contraindications for impacted third molar extraction).

Exclusion criteria: (1) the patient having incomplete data of panoramic radiography or CBCT, (2) the imaging showing root-IAC separation, (3) the patient having his/her tooth partially extracted.

### IMTMs classification

The collection of all the data of panoramic radiography and CBCT and the diagnosis of the impaction features of all IMTMs was conducted separately by two radiologists on the same computer in consensus. When there was disagreement, the final decision was made by consultation with the third radiologist. The three radiologists all had more than 5 years of expertise. The repeatability of both the radiologists who performed the diagnosis was evaluated by Cohen's kappa in SPSS v22. The results of Cohen's kappa for both radiologists were over 0.8.

**Fig. 1:**
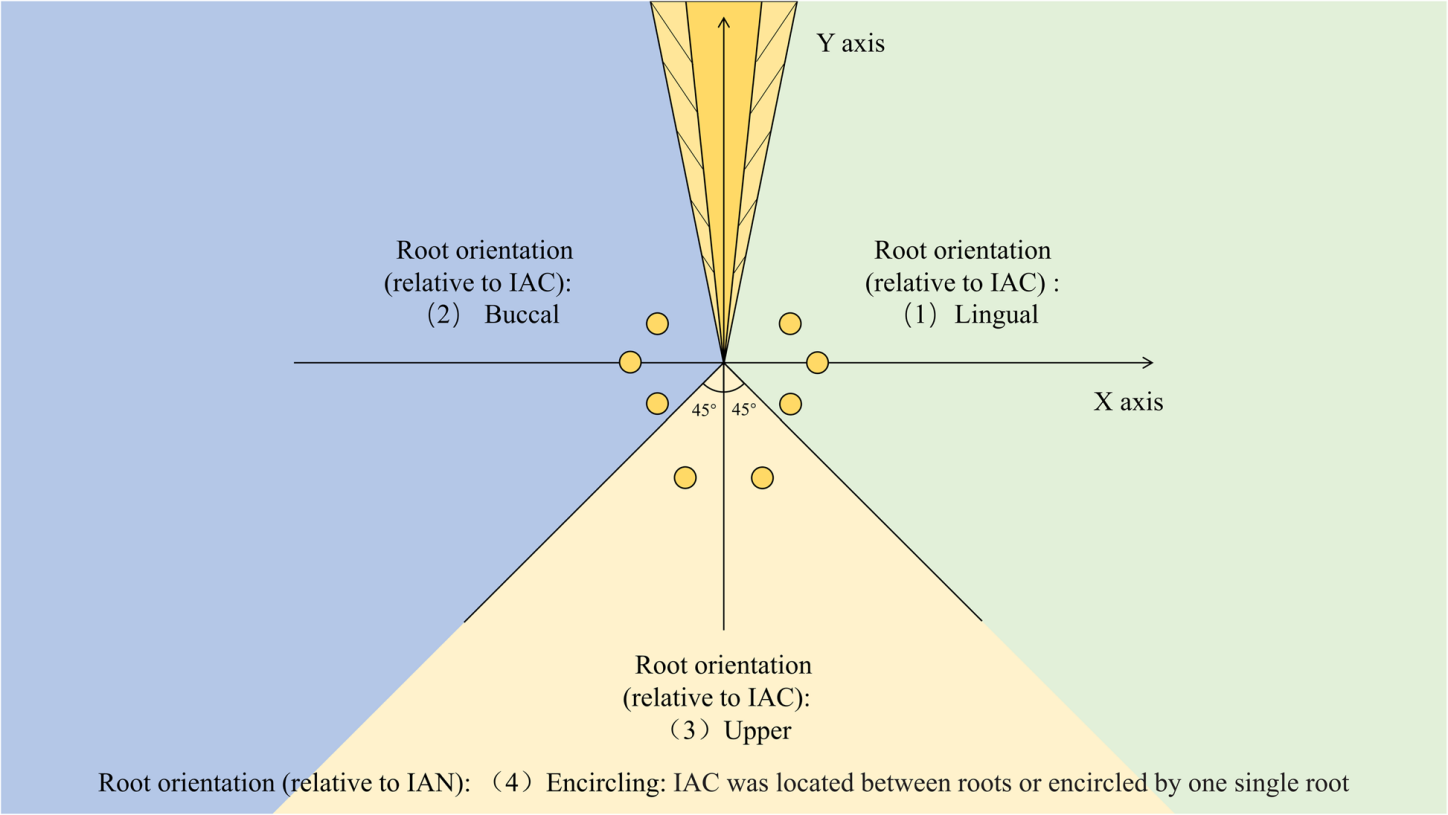
The schematic diagram of coordinate axis and the classification of root orientation

When using CBCT for image analysis, the process of section selection is as follows:

1. Determine the coordinate axis: adjust the shape and size of the dental arch so that the CBCT image can clearly and completely display the mandible and mandibular third molars. Move the intersection point of the CBCT cross observation axis to the root apex, and adjust the angles of the observation axis on the sagittal plane and the coronal plane, so that the longitudinal axis passes through the root axis. The cross intersection point of the adjusted CBCT observation axis is used as the origin of the coordinate axis, the vertical axis is marked as the Y axis, and the horizontal axis is marked as the X axis. On the coronal plane, define the positive Y axis above the root tip, the negative Y axis below the root tip, the positive X axis on the buccal side of the root tip, and the negative X axis on the lingual side of the root tip. And according to the relative position of the IAN and the root, the root orientation is divided into four categories (See Fig. 1).
2. Determine the position of the mandibular canal relative to the impacted mandibular third molar: Move the observation axis in the coronal and sagittal planes to find the section where the relative distance between the mandibular third molar and the mandibular canal is the closest. The coronal plane determines the position of the mandibular third molar root relative to the mandibular canal according to the position in the coordinate system.

**Fig. 2:**
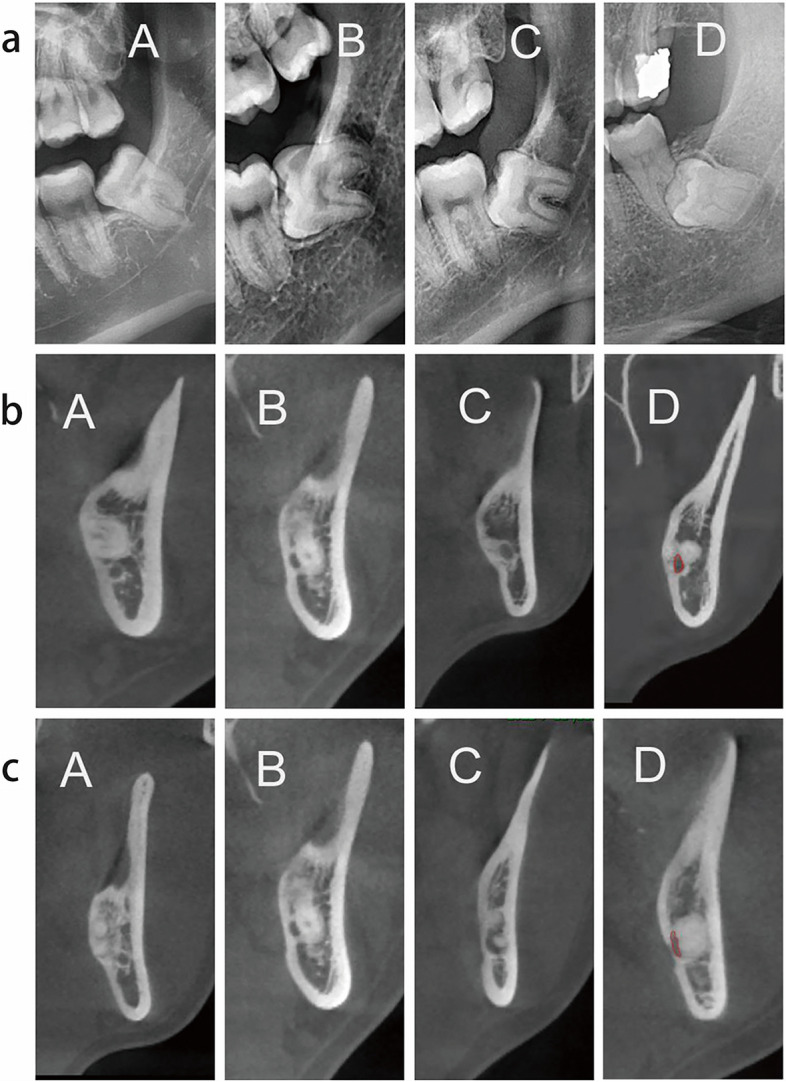
Impaction classification criteria of IMTMs.  **a** A. complete eruption, B. partial eruption, C. mucosal impaction, D. osseous impaction. **b** A. the root was above IAN, B. the root was located on the buccal side of IAN, C. the root was located on the lingual side of IAN, D. IAC was located between roots or encircled by one single root. **c** A. the boundary of IAC was intact (level I), B. the root oppressed IAC, and the boundary of IAC was incomplete (level II), C. the root protruded into IAC, and IAC was squeezed (level III), D. the root protruded into IAC, and IAC was flattened (level IV).

The classification of IMTM is as follows:

1. Classification of impaction depth (Fig. 2a): complete eruption, more than 1/2 of the crown erupted; partial eruption, less than 1/2 of the crown erupted; mucosal impaction, the crown was minimally or partially surrounded by bone without tooth eruption; osseous impaction, the crown was surrounded by bone without tooth eruption.
2. Classification of root orientation [18] (Fig. 2b): upper, the root was above IAC; buccal, the root was located on the buccal side of IAC; lingual, the root was located on the lingual side of IAC; encircling, IAC was located between roots or encircled by one single root.
3. Classification of contact degree of root-IAC [19] (Fig. 2c): level I (adjacent), the root just contacted IAC with its boundary clear; level II (oppressed), the root oppressed IAC with its boundary incomplete; level III (protruding), the root protruded into IAC and IAC was squeezed; level IV (flattening), the root protruded into IAC and IAC was flattened.

### Treatment procedure and outcome

All patients were informed before tooth extraction that IAN injury might occur after tooth extraction and signed a surgical consent form. All IMTMs were extracted by the same senior oral surgeon using the same surgical instruments under local anesthesia. The process is as follows: First, the doctor used articaine plus epinephrine for the inferior alveolar nerve, buccal nerve, and glossal nerve block anesthesia plus local infiltration anesthesia. Second, the gingiva was incised using a triangular flap design and the alveolar bone was removed using a dental handpiece. Then, the third molar was extracted using an elevator and dental forceps. Finally, the tooth extraction wound was irrigated with normal saline and sutured. and the bleeding was stopped by compression with cotton balls. Antibiotics were used after tooth extraction when necessary. When the stitches were removed a week post-operation, IAN-relevant maxillofacial areas were examined by 2-point discrimination, pin-prick, and light touch, to determine whether IAN injury occurred. Patients with numbness of the lower lip were followed up by telephone concerning IAN recovery at 2 weeks, 1 month, 3 months, and 6 months post-operation. The patient with disappeared numbness was asked for a subsequent visit for verification of IAN recovery.

### Statistical analysis

The chi-square / Fisher’s exact test was used to compare the incidence of IAN injury among IMTMs with different impaction depths, root orientation, or contact degree of root-IAC. The correlation between root orientation and impaction depth/contact degree of root-IAC was analyzed based on the Lambda coefficient. The datasets were divided into the buccal group and the non-buccal group in terms of root orientation. Age, sex, impaction depth, and contact degree of root-IAC, were taken as confounding variables. Binary logistic regression was used for modeling. The propensity score of individual cases was calculated by the nearest neighbor matching method with no replacement, and the caliper was set to 0.2. The two groups (buccal and non-buccal) were matched at a ratio of 1:1. The Fisher’s exact test was used to analyze the difference in IAN injury between the two groups before and after PSM, and p < 0.05 was considered statistically significant. All data were analyzed in IBM SPSS Statistics (v22) and R (v4.1.2). To be precise, we used package "CreateTableOne" for baseline data statistics, package "tableone" to calculate the standard mean error (SMD) of each variable, and functions "glm" and "predict" to calculate each case's propensity score (PS). Then package "Matching" was used to match the cases with similar propensity scores at the ratio of 1:1 (caliper set to 0.2 with no replacement). And finally, packages "ggplot2" and "tidyverse" were used for plotting.

## Results

### The overall outcome of IAN injury after IMTMs extraction

In this study, 21 cases of IAN injury with numbness of the lower lip occurred after IMTM extraction. The incidence of IAN injury was 3.90% (21/539). The IMTMs information was shown in Supplementary Table S1.

### Comparison of the incidence of IAN injury among different groups of IMTMs

In terms of impaction depth, except that no IAN injury was found for IMTMs with complete eruption (0/25), the incidence of IAN injury for partial eruption, mucosal impaction, and osseous impaction, was 0.79% (1/127), 2.81% (5/178), and 7.18% (3/209), respectively.

In terms of root orientation, except that no IAN injury was found for the encircling group (0/15), the incidence of IAN injury for the buccal group, upper group, and lingual group was 7.62% (16/210), 1.92% (2/104), and 1.43% (3/210), respectively.

In terms of contact degree of root-IAC, the incidence of IAN injury for level I, II, III, and IV, was 1.18% (2/169), 3.08% (5/130), 4.11% (6/146), and 8.51% (8/94), respectively.

Taken together, there were significant differences in the incidence of IAN injury after IMTM extraction among different types of impaction depth (*p* = 0.006), root orientation(*p* = 0.008), and contact degree of root-IAC (*p* = 0.02). The results are shown in Table 1.

**Table 1:** Comparison of the incidence of IAN injury among different types of IMTMs

Value	Group	All	No IAN injury	IAN injury	*p*
N		539	518	21	
Age (S.D.)		28.94 (7.40)	28.83 (7.26)	31.62 (10.07)	.09
Gender (%)	Female	361(67.0)	347 (67.0)	14 (66.7)	.98
	Male	178(33.0)	171 (33.0)	7 (33.3)	
Orientation (%)	Lingual	210(39.0)	207 (40.0)	3 (14.3)	.008
	Buccal	210(39.0)	194 (37.5)	16 (76.2)	
	Upper	104(19.3)	102 (19.7)	2 (9.5)	
	Encircling	15(2.7)	15 (2.9)	0 (0.0)	
Contact (%)	Level I	166(30.8)	165 (31.9)	1 (4.8)	.02
	Level II	130(24.1)	125 (24.1)	5 (23.8)	
	Level III	149(27.6)	142 (27.4)	7 (33.3)	
	Level IV	94(17.4)	86 (16.6)	8 (38.1)	
Depth (%)	Complete eruption	25(4.6)	25 (4.8)	0 (0.0)	.006
	Partial eruption	127(23.6)	126 (24.3)	1 (4.8)	
	Mucosal impaction	178(33.0)	174 (33.6)	4 (19.0)	
	Osseous impaction	209(38.8)	193 (37.3)	16 (76.2)	

### Correlation analysis of root orientation with impaction depth/root orientation

As demonstrated above, IMTMs with the root located on the buccal side of IAC were more prone to IAN injury. However, it was unclear whether the difference was attributed to the distribution unevenness of impaction depth and contact degree of root-IAC among different groups of root orientation. Therefore, the chi-square test of the contingency table was used to analyze the correlation between root orientation and impaction depth/contact degree of root-IAC, and the lambda coefficient was 0.276(*p* < 0.001) and 0.102(*p* = 0.003), respectively (the results are shown in Table 2). The proportion of level III and IV of contact degree in the buccal group was 70.1% (149/210), which was higher than that of 28.6% in the non-buccal group (94/329). Furthermore, the proportion of mucosal and osseous impaction in the buccal group was 80.5% (169/210), which was higher than that of 66.3% in the non-buccal group (218/329).

**Table 2:** Correlation analysis of root orientation and impaction depth / contact degree of root-IAC

		Orientation						
		Upper	Buccal	Lingual	Encircling	Total	Lambda coefficient	*p*
Contact	Level I	25	16	125	0	167	0.276	< .001
	Level II	38	45	43	4	129		
	Level III	39	69	37	4	149		
	Level IV	2	80	5	7	94		
Depth	Complete eruption	1	6	18	0	25	0.102	.003
	Partial eruption	18	35	71	3	127		
	Mucosal impaction	35	70	69	4	178		
	Osseous impaction	50	99	52	8	209		

The correlation analysis revealed the necessity of reasonable statistical methodologies to balance the distribution of impaction depth, contact degree of root-IAC, and other confounding factors, among different groups of root orientation, for a better insight into the independent effect of root orientation on IAN injury.

### PSM of the buccal and non-buccal type of root orientation

**Fig. 3:**
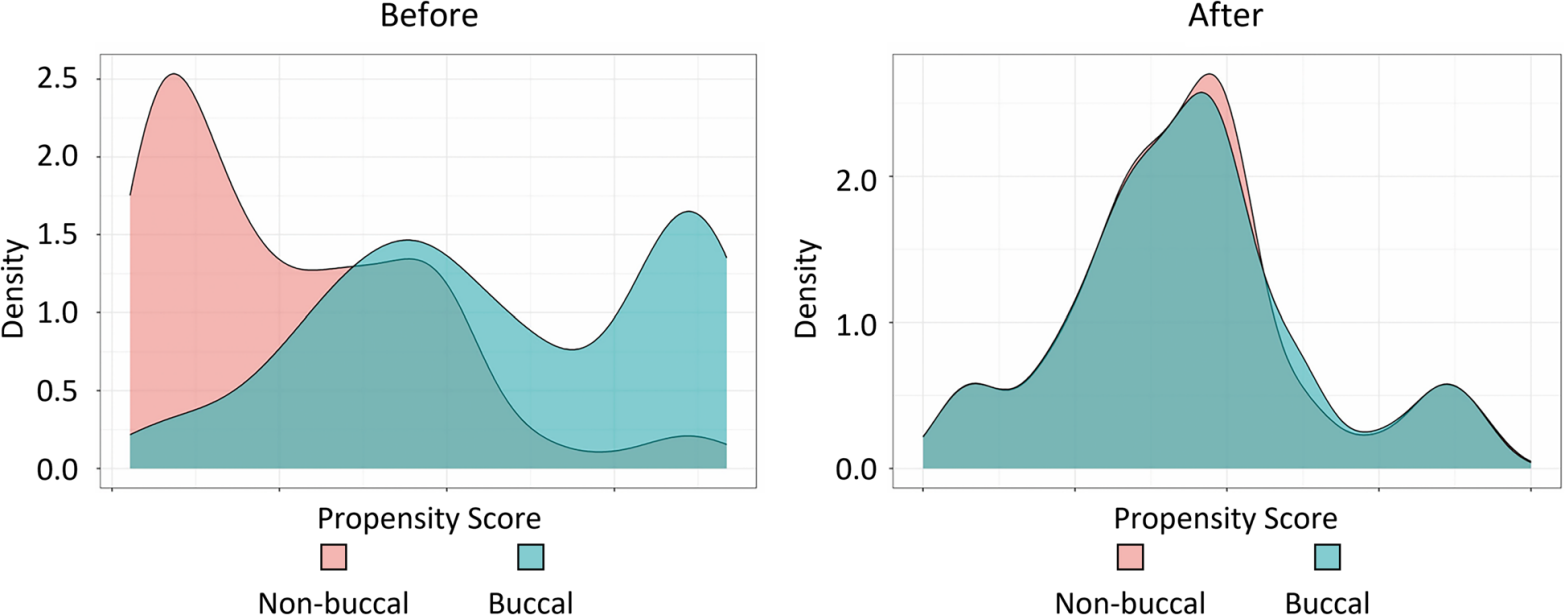
Kernel density of the PS score before and after PSM

Age, sex, impaction depth, and contact degree of root-IAC were considered confounding variables. Binary logistic regression was used to model and calculate the propensity score of each case. Using nearest neighbor matching with no replacement and setting the caliper to 0.2, the buccal group and non-buccal group were matched at the ratio of 1:1. There were 21 cases with IAN injury and 518 cases without IAN injury before PSM and 9 cases with IAN injury and 257 cases without IAN injury after PSM. The case number of buccal/non-buccal was 210/329 before matching and 133/133 after matching. The standard mean difference (SMD) of age, sex, impaction depth, and contact degree of root-IAC, was respectively 0.066, 0.457, 0.357, and 1.245 before PSM, and was respectively 0.05, 0.051, 0.122, and 0.101 after PSM, as shown in Table 3. It was demonstrated that PSM significantly reduced the interference of confounding factors. In addition, the plot of the Kernel density of the PS score before and after PSM was shown in Fig. 3, indicating that group-to-group comparability was significantly improved after PSM.

**Table 3:** Distribution of confounding variables before and after PSM

	Unmatched			Matched		
	Buccal	Non-buccal	SMD	Buccal	Non-buccal	SMD
N	210	329		133	133	
Age (S.D.)	28.64 (7.31)	29.13 (7.46)	0.066	28.41 (7.65)	28.79 (7.27)	0.05
Gender-male (%)	43 (20.5)	135 (41.0)	0.457	35 (26.3)	38 (28.6)	0.051
Depth (%)			0.357			0.122
Complete eruption	6 (2.9)	19 (5.8)		6 (4.5)	5 (3.8)	
Partial eruption	35 (16.7)	92 (28.0)		23 (17.3)	25 (18.8)	
Mucosal impaction	70 (33.3)	108 (32.8)		45 (33.8)	51 (38.3)	
Osseous impaction	99 (47.1)	110 (33.4)		59 (44.4)	52 (39.1)	
Contact (%)			1.245			0.101
Level I	16 (7.6)	150 (45.6)		16 (12.0)	12 (9.0)	
Level II	45 (21.4)	85 (25.8)		45 (33.8)	48 (36.1)	
Level III	69 (32.9)	80 (24.3)		58 (43.6)	59 (44.4)	
Level IV	80 (38.1)	14 (4.3)		14 (10.5)	14 (10.5)

### The effect of root orientation on IAN injury before and after PSM

Before PSM, the incidence of IAN injury was 7.62% in the buccal group and 1.52% in the non-buccal group, and the difference was statistically significant (*p* <.001). The odds ratio (OR) was 5.344 and the risk ratio (RR) was 5.013. After PSM, the incidence was 6.02% in the buccal group and 0.75% in the non-buccal group, and the difference was statistically significant (*p*=.04), as shown in Table 4. It is evident that after balancing confounding factors, the risk of IAN injury of IMTMs whose root was located on the buccal side of IAN was significantly higher than that of non-buccal counterparts. The odds ratio (OR) was 8.448 and the risk ratio (RR) was 8.

**Table 4:** Comparison of the incidence of IAN injury before and after PSM

Orientation	Unmatched				Matched			
	No IAN injury	IAN injury	Injury ratio	*p*	No IAN injury	IAN injury	Injury ratio	*p*
Buccal	194	16	7.62%	< .001	125	8	6.02%	.04
Non-buccal	324	5	1.52%		132	1	0.75%	
Total	518	21	3.90%		257	9	3.38%	

## Discussion

There is no consensus about the effect of IMTMs root orientation on inferior alveolar nerve injury. Using propensity score matching, we found that the third molar whose root was located on the buccal side of the inferior alveolar nerve had a higher risk of IAN injury than other groups.

IAN injury is one of the most serious complications after IMTM extraction, which generally brings a heavy psychological burden to patients. The causes of IAN injury are related to the operator's experience, the surgical method, and the impaction characteristics of IMTMs such as impaction depth, contact degree of root-IAC, and so on [20, 21]. Prediction of the risk of IAN injury necessitates reasonable imaging technologies with an accurate assessment of the impaction characteristics of IMTMs. In this study, panoramic radiography and CBCT examination were both used for all the patients involved. CBCT allows 3D observation of IMTMs and IAC, and measurement of root-IAC contact and mandibular morphology with an accuracy of 0.1 mm [22].

In this study, 21 cases of IMTMs ended up with IAN injuries. These teeth had the following features: (1) deep impaction (1 of partial eruption, 5 of mucosal impaction, and 15 of osseous impaction), (2) close contact with IAC (5 of level II, 6 of level III, and 8 of level IV), (3) most of the roots located on the buccal side of IAC (16 on the buccal side, 2 on the upper side, and 3 on the lingual side).

The chi-square / Fisher’s exact test showed that impaction depth, contact degree of root-IAC, and root orientation, all significantly influenced the incidence of IAN injury, which was generally in accordance with previous literature [16, 23, 24]. The effects and causes of impaction depth and contact degree of root-IAC have been recognized. For example, deep-impacted IMTMs are generally associated with poor visualization and access, more complex operation, and longer operation time, which increases the incidence of IAN injury. However, the effect of root orientation on the incidence of IAN injury is inconclusive. Although some previous studies suggested that the root located on the buccal side of IAC was associated with a higher risk of IAN injury [25, 26], other studies suggested that the effect of root orientation was not statistically significant [7, 8].

Since previous studies were mostly retrospective and observational, the inconsistent conclusions concerning root orientation may be attributed to the disturbance of various confounding factors. In this study, we found that root orientation was correlated with impaction depth and contact degree of root-IAC, and the root located on the buccal side of IAC tended to be accompanied by close contact of root-IAC and deep impaction, which was rarely mentioned in the previous literature.

Reasonable statistical methodologies are necessary to further clarify the influence of root orientation. In this study, the PSM method was used to match the buccal and non-buccal groups at 1:1, for reducing the interference of age, sex, impaction depth, and contact degree of root-IAC. Compared to regression-based traditional statistical strategies for controlling confounding factors in observational studies, the advantage of PSM is that it is minimally limited by the number of events, and its efficacy is warranted especially when the number of confounders is large or the number of outcomes is small [27], which is highly applicable to our study.

It was shown that the SMD value of the four confounding factors after PSM all decreased significantly, indicating that group-to-group comparability was significantly improved. The effect of root orientation was further analyzed after PSM by Fisher’s exact test. It was shown that the buccal group had a higher risk of IAN injury than the non-buccal group, and the OR value was 8.448. Therefore, we believe that root orientation is an independent risk factor of IAN injury after extraction of IMTMs, and the root located on the buccal side of IAN is more vulnerable to IAN injury. As a possible explanation, the root located on the buccal side is more likely to move downward or lingually during operation, and finally squeeze IAN, because the force of the operator is generally from the buccal side, and there are cancellous bones on the lingual side.

The main limitation of this study is that although we include four important covariables, such as age, sex, impaction depth, and contact degree for propensity score matching, however, there is a potential risk of results being confounded by other variables that are not included in this study (such as the duration and procedure of the operation). It is noteworthy that there was no IAN injury in all 15 cases of the encircling type of root orientation. Intuitively, the risk of the encircling type is greater than that of the buccal type. As a possible explanation, the incidence of the encircling type of root orientation is relatively low so the sample size is relatively small in this study, which makes it inaccurate to analyze the influence of the encircling type.

## Conclusions

In conclusion, this study suggests that root orientation is an independent risk factor of IAN injury after extraction of IMTMs, and the root located on the buccal side of IAC is associated with a higher risk than non-buccal counterparts, which may be a new indicator for reasonable prediction of IAN injury. Future studies are expected to involve more cases of the encircling type of root orientation for comparison with the buccal type, in order to provide a better insight into the influence of root orientation.

## Supplementary Information

**Additional file 1. **Supplementary tables

## Data Availability

The datasets generated and analyzed during the current study are publicly available in supplementary tables.

## Abbreviations

PSM: Propensity score matching
IMTMs: Impacted mandibular third molars
IAC: Inferior alveolar canal
IAN: Inferior alveolar nerve
CBCT: Cone beam computed tomography
OR: Odds ratio
