# Correction: The effect of root orientation on inferior alveolar nerve injury after extraction of impacted mandibular third molars based on propensity score‑matched analysis: a retrospective cohort study

**DOI:** 10.1186/s12903-024-05219-0

**Published:** 2024-11-23

**Authors:** Yuhao Liu, Weijie Zhuang, Kechen Li, Wenbin Yang, Yihong Tian, Shijun Kuang

**Affiliations:** 1 Hospital of Stomatology, Guangdong Provincial Clinical Research Center of Oral Diseases, Guangdong Provincial Key Laboratory of Stomatology, Guangdong Key Laboratory for Dental Disease Prevention and Control, Sun Yat-Sen University, No.56, Lingyuan West Road, Yuexiu District, Guangzhou City, Guangdong Province 510030 China; 2 State Key Laboratory of Oral Diseases, National Clinical Research Center for Oral Diseases, West China Hospital of Stomatology, Sichuan University, No.14, Third Section of Ren Min Nan Road, Chengdu City, Sichuan Province 610041 China

**Correction** **:** **BMC Oral Health 23, 929 (2023)**

**https://doi.org/10.1186/s12903-023-03661-0**

In this article [1], the order of the authors in the author group is incorrect. The incorrect and correct author group is listed in this correction.

Incorrect order:

Shijun Kuang, Yuhao Liu, Weijie Zhuang, Kechen Li, Wenbin Yang and Yihong Tian

Correct order:

Yuhao Liu, Weijie Zhuang, Kechen Li, Wenbin Yang, Yihong Tian, and Shijun Kuang

## References

[1] Kuang S, Liu Y, Zhuang W, Li K, Yang W, Tian Y. The effect of root orientation on inferior alveolar nerve injury after extraction of impacted mandibular third molars based on propensity score-matched analysis: a retrospective cohort study. BMC Oral Health. 2023;23(1):929. 38008723 10.1186/s12903-023-03661-0 PMC10680265

